# Effect of location of opening incision on astigmatic correction after small-incision lenticule extraction

**DOI:** 10.1038/srep35881

**Published:** 2016-10-24

**Authors:** Tommy C. Y. Chan, Alex LK Ng, George P. M. Cheng, Zheng Wang, Victor C. P. Woo, Vishal Jhanji

**Affiliations:** 1 Hong Kong Eye Hospital, Kowloon, Hong Kong; 2 Department of Ophthalmology and Visual Sciences, The Chinese University of Hong Kong, Kowloon, Hong Kong; 3 Department of Ophthalmology, The University of Hong Kong, Pok fu lam, Hong Kong; 4 Hong Kong Laser Eye Center, Tsim Sha Tsui, Hong Kong; 5 Zhongshan Ophthalmic Center, Sun Yat-sen University, Guangzhou, China

## Abstract

We compared the visual and refractive outcomes between 2 different incisional sites in small incision lenticule extraction (SMILE) for low myopic astigmatism. This was a contralateral eye study. Consecutive cases that underwent bilateral SMILE surgery were included. Procedures for both eyes were identical apart from the location of opening incision. The incision was set on the temporal side for the right eye (Group 1), while a superior incision was set for the left eye (Group 2). Twenty-nine patients with a mean age of 35.0 ± 9.6 years were included. Preoperative visual and refractive parameters were comparable between the 2 groups (p > 0.250). At 3 months, the logMAR uncorrected distance visual acuity was 0.074 ± 0.090 in Group 1 and 0.084 ± 0.130 in Group 2 (p = 0.861). No difference was found in the postoperative manifest spherical equivalent (p = 0.501) and manifest cylinder (p = 0.178) between the 2 groups. The efficacy index was 0.85 ± 0.16 in Group 1 and 0.85 ± 0.20 in Group 2 (p = 0.828). Astigmatic correction was not significantly affected by the location of opening incisions using vector analysis. Our study did not find significant differences in visual and refractive outcomes with temporal or superior opening incision during SMILE surgery.

Femtosecond laser has been used to create thin and uniform flaps in laser assisted *in-situ* keratomileusis (LASIK) with great precision^1^,^2^. The use of femtosecond laser has recently been extended to a new form of corneal refractive procedure termed refractive lenticule extraction. According to the intended refractive correction, a thin lenticule was cut with femtosecond laser and was subsequently removed. Small-incision lenticule extraction (SMILE) represents the technique in which the lenticule is removed through the creation of one or more small peripheral corneal incisions^3^,^4^. Various studies have demonstrated that SMILE is safe and effective for corneal refractive correction of myopia and astigmatism^5^,^6^,^7^,^8^,^9^.

Treatment parameters vary amongst studies utilizing SMILE for myopic correction^10^. Scanning trajectory of the femtosecond laser has been shown to affect early visual recovery and refractive outcomes after SMILE^11^. On the other hand, visual performance and optical quality were not affected by energy settings of the femtosecond laser^12^. The peripheral corneal incision, through which the lenticule is extracted, was also not standardized. More than one opening incisions have been used^3^,^6^, while most studies had the lenticule extracted through a single superior opening^4^,^5^,^11^,^13^,^14^. The size of the incision also varied amongst or within studies^6^,^7^,^13^. To our knowledge, no studies have tried to investigate the effect of incisional location on the refractive outcomes of SMILE. The purpose of this study aims to compare the visual and refractive outcomes between 2 different incisional sites in SMILE for low myopic astigmatism. Low cylinder correction was chosen because the opening incision is usually only 100 to 140 μm in depth^10^, its potential effect could be masked by a high magnitude of astigmatic correction.

## Results

This was a contralateral eye comparative study. Twenty-nine patients with a mean age of 35.0 ± 9.6 years were included. The peripheral incision was located at the temporal cornea for the right eye (Group 1) and at the superior corneal for the left eye (Group 2) for each patient. There was no significant difference in manifest spherical equivalent (p = 0.279), manifest sphere (p = 0.250), manifest cylinder (p = 0.465) and uncorrected distance visual acuity (CDVA) (p = 1.000) between the 2 groups preoperatively (Table 1). All surgeries were uneventful without any intraoperative complications.

**Figure 1:**
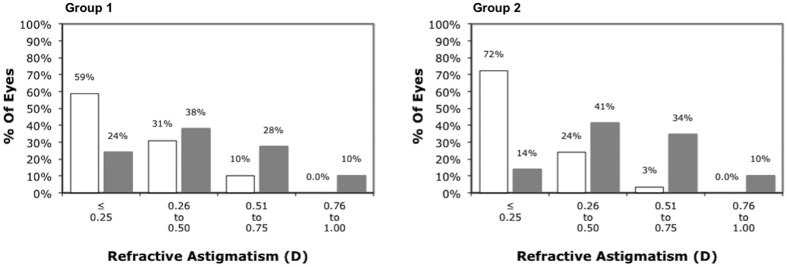
Distribution of refractive astigmatism in diopters (**D**) before (grey) and after (white) small-incision lenticule extraction with a temporal (Group 1) or superior (Group 2) opening incision.

At 3 months, the logMAR corrected distance visual acuity (CDVA) was 0.015 ± 0.029 in Group 1 and 0.012 ± 0.032 in Group 2 (p = 0.564). The logMAR UDVA was 0.074 ± 0.090 in Group 1 and 0.084 ± 0.130 in Group 2 (p = 0.861). No significant difference was found in the postoperative manifest spherical equivalent (p = 0.501), manifest sphere (p = 0.910) and manifest cylinder (p = 0.178) between the 2 groups (Table 1). Seventeen (58.6%) eyes in Group 1 were with ± 0.25 Diopter (D) of the attempted cylindrical correction at 3 month. The corresponding value in Group 2 was 21 (72.4%) eyes (p = 0.269) (Fig. 1).

The efficacy index, which was calculated as the ratio of postoperative UDVA over preoperative CDVA, was 0.85 ± 0.16 in Group 1 and 0.85 ± 0.20 in Group 2 (p = 0.828). The safety index, which was determined as the ratio of postoperative CDVA over preoperative CDVA, was 0.96 ± 0.08 in Group 1 and 0.97 ± 0.07 in Group 2 (p = 0.799). No postoperative corneal complication, such as wound dehiscence, inflammation and infection, was observed in any patient.

### Vector analysis

**Figure 2:**
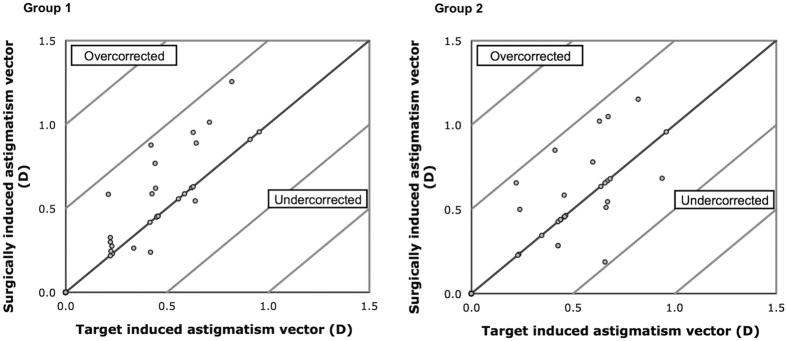
Surgically induced astigmatism vector versus target induced astigmatism vector in diopters (**D**) after small-incision lenticule extraction with a temporal (Group 1) or superior (Group 2) opening incision.

**Figure 3:**
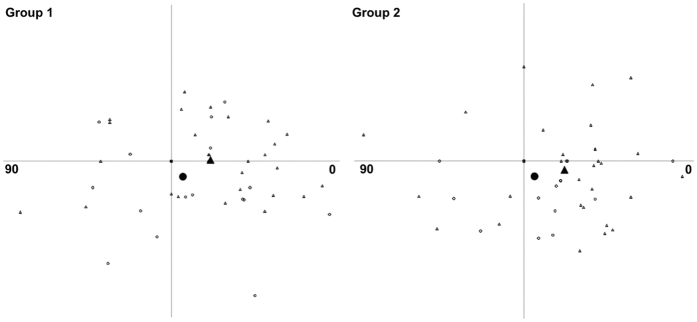
Double-angle plots for preoperative target induced astigmatism (open triangle) and postoperative difference vector after small-incision lenticule extraction. The filled triangle represents the average preoperative target induced astigmatism; the filled circle represents the average postoperative difference vector at 3 months.

The vector analysis results using the 3-month refractive data are shown in Table 2. There was no significant difference in the arithmetic mean of target induced astigmatism (TIA), surgically induced astigmatism (SIA), difference vector (DV) and magnitude of error (ME) between Group 1 and 2 (p > 0.204). Scatterplots of SIA versus TIA for both groups are shown in Fig. 2. The arithmetic mean of AE and absolute mean of AE were similar between Group 1 and 2 (p > 0.534). The double-angle plots for preoperative TIA and postoperative DV for Group 1 and 2 are shown in Fig. 3. The centroid coordinates (x, y) of TIA were (0.23 ± 0.42, 0.01 ± 0.22) for Group 1 and (0.24 ± 0.42, −0.06 ± 0.31) for Group 2, indicating the average astigmatism was with-the-rule preoperatively. At 3 months after surgery, the centroid coordinates (x, y) of DV were (0.07 ± 0.30, −0.10 ± 0.26) for Group 1 and (0.06 ± 0.24, −0.10 ± 0.16) for Group 2. The centroid of both groups moved closer to the origin signifying a reduction in cylinder value.

At 3 months, the correction index (CI), defined as the ratio of SIA to TIA, was 1.17 (1.14 to 1.20) in Group 1 and 1.05 (1.02 to 1.09) in Group 2. Index of success (IS), defined as DV divided by TIA, was 0.38 (0.32 to 0.44) in Group 1 and 0.19 (0.14 to 0.25) in Group 2. Flattening index (FI), defined as ratio of the amount of astigmatism reduction achieved by the effective proportion of the SIA at the intended meridian to TIA, was 0.75 (0.69 to 0.80) in Group 1 and 0.85 (0.78 to 0.92) in Group 2.

## Discussion

Our results showed that there was no clinical significance between the temporal and superior incision in the visual and refractive outcomes after SMILE for low astigmatism. The efficacy index was 0.85 for both groups in the current study. Both groups were equally safe with a safety index over 0.96. The corresponding efficacy and safety index was 0.90 and 1.07 at 3 months of a prospective analysis of 670 post-SMILE eyes^16^.

Since the first SMILE performed by Sekundo and colleagues using 2 opposing 5.0 mm incisions, there has not been any standard size and location for the peripheral incision^3^. Sekundo *et al*. treated half of the eyes with the opening incisions at the superior and inferior cornea and the other half at the nasal and temporal position. However, no direct comparison between the 2 sets of incisions was performed^3^. Majority of studies in the literature used a single superior incision with a side-cut length of 2.0 to 5.0 mm (30- to 60-degree)^4^,^5^,^11^,^13^,^14^. Shah and colleagues demonstrated that early visual recovery and refractive outcomes after SMILE were affected by the scanning trajectory of the laser^11^. In the study, most cases had a superior incision of 4.0 to 5.0 mm (50-degree). Some cases had either a nasal or temporal incision, but comparison among incisional positions was not carried out^11^. On the other hand, two 2-mm incisions were created in all eyes with one superonasally and the other superotemmporally in a retrospective study of SMILE in low myopia^6^. In the current study, a 2-mm opening incision was created superiorly or temporally.

Analyzing the astigmatic effect by simply comparing the preoperative and postoperative average cylinder values may not be sufficient. Vector analysis can generally describe the process of astigmatism correction focusing on both magnitude and direction. The Alpins method was chosen for the current study^15^. Using vector analysis, astigmatic correction for eyes with low preoperative cylinder was not significantly affected by the location of opening incisions. The flattening effect was similar (FI: 0.75 to 0.85), but there was slight overcorrection (CI: 1.05 to 1.17) in both groups. Previous study using vector analysis showed an astigmatic overcorrection for eyes with a preoperative cylinder of 0.25 to 0.50 D after LASIK^17^. On the other hand, Ivarsen *et al*. noted undercorrection of astigmatic error after SMILE for preoperative cylinder of 0.75 D or more, and there was a minor induced torsion in eyes corrected for low astigmatism^4^. We also observed a slight rotation of cylinder axis (−6.9 degrees) in eyes with a temporal opening incision although this was not statistically different from eyes with a superior incision (−0.39 degrees). Despite a lower success of astigmatic correction (higher IS) was found in eye with temporal incision (0.38 versus 0.19), the difference in ME between the 2 incisional locations was very minimal (0.05 D), and was clinical and statistically insignificant. Indeed, it has been showed that lifting the flap in femtosecond lenticule extraction had a similar astigmatic effect compared to SMILE^9^. The result further suggested that the location of opening incision in SMILE has minimal astigmatic effect.

We observed that the absolute AE was very similar between temporal and superior incision. However, it showed a less favorable range (29 degrees), suggesting variable factors such as healing or alignment at an individual level. Alpins noted that the proportion of loss of flattening effect is 1.5% when treatment is misaligned by 5 degrees, 13.4% when 15 degrees, and as high as 50% when 30 degrees. The effect is lost totally when the treatment is misaligned by 45 degrees^18^. This emphasizes the importance of treatment alignment for successful correction of astigmatism, in addition to the treatment magnitude. This is particularly noticeable when the amount of astigmatic correction is low because the measurement error tended to become relatively obvious^8^,^19^.

The current study was limited by its small sample size and non-randomized design. We did not measure corneal topography postoperatively that precluded vector analysis of topographic astigmatism. Furthermore, contrast sensitivity and higher-order aberration after SMILE were shown to be superior compared to LASIK^20^. Future studies are warranted to compare the visual quality between different locations of SMILE opening incision. Nevertheless, it is the first study that compares the location of opening incision for SMILE. This study demonstrates that both SMILE procedures, with temporal or superior opening incision, offered favorable results in the correction of low myopic astigmatism. No significant difference in visual and refractive outcomes between the 2 incisions was found.

## Methods

This study was part of a prospective audit of consecutive patients who received SMILE between January 1, 2014 and December 31, 2014 at the Hong Kong Laser Eye Center, Hong Kong. The Joint Chinese University of Hong Kong-New Territories East Cluster Clinical Research Ethics Committee approved the study protocol. An informed consent was obtained from all participants. The study adhered to the tenets of the Declaration of Helsinki.

All patients underwent a complete ophthalmic examination and had no ocular abnormality except myopia and myopic astigmatism with a corrected distance visual acuity (CDVA) of 20/20 or better in both eyes. CDVA was measured using a Snellen chart at 6 meters in a well-illuminated room. Patients with a stable refraction for more than 1 year, myopia of ≥1.0 Diopter (D) and corneal astigmatism between 0.25 D and 1.00 D in both eyes were included. All eyes had emmetropia as the target refraction. Only patients who had undergone SMILE for both eyes in the same operation session were included. Patients with corneal thickness of <500 μm, suspicion of keratoconus on corneal topography (displacement of the corneal apex, decrease in thinnest-point pachymetry, asymmetric topographic pattern), ocular inflammation, and infection were exclusion criteria for SMILE.

Preoperative examinations included slit-lamp biomicroscopy, dilated funduscopy examinations, uncorrected distance visual acuity (UDVA), CDVA, manifest refraction and corneal topography (Orbscan IIz, Bausch & Lomb, Rochester, NY, USA).

### Surgical Technique

The same surgeon (G.C.) performed all the surgery under topical anesthesia. SMILE was performed using the 500-kHz VisuMax femtosecond laser (Carl Zeiss Meditec, Jena, Germany) using an established technique^3^. The following parameters were used: cap thickness, 120 μm; cap diameter, 7.5mm; lenticule diameter, 6.5 mm with a transition zone of 0.1 mm; cut energy, 1.4 μJ; spot and tracking distance, 2.0–3.0 μm. The back of the intrastromal lenticule was created from periphery to center of the cornea. The anterior lamellar cut was subsequently created from center to periphery of the cornea, which extended toward the surface to create a 2-mm incision, from which the stromal lenticule was extracted. The parameters for both eyes were identical apart from the location of the peripheral incision. The peripheral incision was set on the temporal side for the right eye (Group 1), while a superior incision was set for the left eye (Group 2). A thin blunt spatula was used to separate the lenticule, which was then grasped with a pair of forceps and removed. The corneal interface was flushed with balanced salt solution.

Postoperatively, all patients received topical tobramycin 0.3% and dexamethasone 0.1% ophthalmic suspension four times a day for 1 week. Preservative free artificial teardrops were used for 3 months postoperatively.

Measurements at postoperative 3 months including uncorrected distance visual acuity (UDVA), corrected distance visual acuity (CDVA) and manifest refraction were analyzed in the current study.

### Statistical Analysis

Refractive astigmatism at the spectacle plane was converted to the corneal plane using vertex distance of 12 mm. It was then analyzed using vector analysis, with consideration of the change in the astigmatic axis, measuring 3 vectors and relationships among them^15^. The target induced astigmatism vector (TIA), defined as the astigmatic change which the surgery was intended to induce; the surgically induced astigmatism vector (SIA), defined as the astigmatic change which the surgery actually induced; and the difference vector (DV), defined as the induced astigmatic change that would enable the initial surgery to achieve its intended target or the postoperative astigmatism. Magnitude of error (ME) is the arithmetic difference between the SIA and TIA. Angle of error (AE) is the angle between the axis of the SIA and TIA.

Statistical analysis was performed using PASW software version 18.0 (SPSS/IBM Inc., Chicago, IL, USA). Wilcoxon signed rank and Chi-square test were used to compare continuous and categorical variables, respectively. A P value less than 0.05 was defined as statistically significant. Indexes of vector analysis were expressed as geometric mean with 95% confidence interval showed within brackets.

## Additional Information

**How to cite this article**: Chan, T. C. Y. *et al*. Effect of location of opening incision on astigmatic correction after small-incision lenticule extraction. *Sci. Rep.* **6**, 35881; doi: 10.1038/srep35881 (2016).

## Figures and Tables

**Table 1:** Preoperative and postoperative characteristics of patients undergoing small-incision lenticule extraction with a temporal (Group 1) or superior (Group 2) opening incision.

	Group 1 (N = 29)	Group 2 (N = 29)	P value^b^
Preoperative			
Corrected Distance Visual Acuity^a^	−0.003 ± 0.015	−0.003 ± 0.015	1.000
Sphere	−5.23 ± 1.91	−4.66 ± 1.92	0.250
Cylinder	−0.55 ± 0.23	−0.59 ± 0.22	0.465
Spherical Equivalent	−5.50 ± 1.91	−4.96 ± 1.93	0.279
Postoperative			
Corrected Distance Visual Acuity^a^	0.015 ± 0.029	0.012 ± 0.032	0.564
Uncorrected Distance Visual Acuity^a^	0.074 ± 0.090	0.084 ± 0.130	0.861
Sphere	−0.021 ± 0.310	−0.030 ± 0.605	0.910
Cylinder	−0.28 ± 0.31	−0.20 ± 0.24	0.178
Spherical Equivalent	−0.16 ± 0.32	−0.13 ± 0.60	0.501

^a^In logarithm of minimum angle of resolution scale.

^b^Comparison between Group 1 and Group 2. P < 0.05 represents statistically significant.

**Table 2:** Vector analysis of astigmatic correction at 3 month after small-incision lenticule extraction with a temporal (Group 1) or superior (Group 2) opening incision using Alpins method.

		Group 1 (N = 29)	Group 2 (N = 29)	P value^b^
TIA	Arithmetic mean ± SD (Diopters)	0.48 ± 0.21	0.53 ± 0.20	0.665
	Range^a^ (Diopters)	0.47 to 0.50	0.52 to 0.54	
	Vector mean	0.23 @ 0.8°	0.25 @−6.9°	
SIA	Arithmetic mean ± SD (Diopters)	0.57 ± 0.28	0.57 ± 0.25	0.991
	Range^a^ (Diopters)	0.56 to 0.59	0.56 to 0.59	
	Vector mean	0.20 @ 16.5°	0.18 @ 6.5°	
DV	Arithmetic mean ± SD (Diopters)	0.27 ± 0.31	0.20 ± 0.24	0.204
	Range^a^ (Diopters)	0.25 to 0.29	0.18 to 0.21	
	Vector mean	0.12 @−28.0°	0.12 @−28.9°	
ME	Arithmetic mean ± SD (Diopters)	0.09 ± 0.17	0.04 ± 0.21	0.458
	Range^a^ (Diopters)	0.08 to 0.10	0.03 to 0.06	
AE	Arithmetic mean ± SD (Degrees)	−6.9 ± 42.2	−0.39 ± 44.3	0.534
	Range^a^ (Degrees)	−9.7 to−4.0	−3.4 to 2.6	
Absolute AE	Arithmetic mean ± SD (Degrees)	29.8 ± 30.2	29.1 ± 32.9	0.869
	Range^a^ (Degrees)	27.7 to 31.8	26.9 to 31.3	
CI	Geometric mean ± SD	1.17 ± 0.43	1.05 ± 0.52	/
	Range^a^	1.14 to 1.20	1.02 to 1.09	
CA	Geometric mean ± SD	0.86 ± 0.27	0.95 ± 0.55	/
	Range^a^	0.84 to 0.87	0.91 to 0.99	
FI	Geometric mean ± SD	0.75 ± 0.81	0.85 ± 1.05	/
	Range^a^	0.69 to 0.80	0.78 to 0.92	
IOS	Geometric mean ± SD	0.38 ± 0.95	0.19 ± 0.83	/
	Range^a^	0.32 to 0.44	0.14 to 0.25	

AE = angle of error; CA = coefficient of adjustment; CI = correction index; DV = difference vector; FI = flattening index; IOS = index of success; SIA = surgically induced astigmatism; SD = standard deviation; TIA = target-induced astigmatism.

^a^95% confidence interval.

^b^Comparison between Group 1 and Group 2. P < 0.05 = statistically significant.
